# An equivalence study: Are patient-completed and telephone interview equivalent modes of administration for the EuroQol survey?

**DOI:** 10.1186/s12955-017-0596-x

**Published:** 2017-01-23

**Authors:** R. Chatterji, J. M. Naylor, I. A. Harris, E. Armstrong, E. Davidson, R. Ekmejian, J. Descallar

**Affiliations:** 1 South Western Sydney Clinical School, UNSW Australia, Liverpool, NSW Australia; 2 Liverpool Hospital Orthopaedic Department, South Western Sydney Local Health District, Liverpool, NSW Australia; 3 Whitlam Orthopaedic Research Centre, Ingham Institute for Applied Medical Research, 1 Campbell St, Liverpool, NSW Australia; 4 Nepean Hospital Physiotherapy Department, Nepean Blue Mountains Local Health District, Penrith, NSW Australia; 5 Ingham Institute for Applied Medical Research, 1 Campbell St, Liverpool, NSW Australia; 6 Orthopaedic Department, Liverpool Hospital, Locked Bag 7103, Liverpool, BC 1871 Australia

**Keywords:** Hip arthroplasty, Knee arthroplasty, Health related quality of life, Telephone, Questionnaire, Equivalence, Mode of administration

## Abstract

**Background:**

To determine if the EuroQol Health Related Quality of Life survey produces equivalent results when administered by phone interview or patient-completed forms.

**Methods:**

People awaiting hip or knee arthroplasty at a major metropolitan hospital participated. They were randomly assigned to receive the EuroQol Health Related Quality of Life survey via telephone, followed by a patient completed form 1 week later, or vice versa. Equivalence was determined using two one-sided tests (TOST) based on minimal clinically-important differences for the visual analogue scale (VAS) and the summary Utility Index. Cohen’s Kappa scores were computed to determine agreement for the individual EuroQoL Likert scale items.

**Results:**

Seventy-six from 90 (84%) participants completed the survey twice. Based on limits set at ±7 and ±0.11 for the VAS and Utility Index, respectively, equivalence was established between the two methods of administration for both the VAS (mean difference 0.05 [90% CI −3.76–3.67]) and the Utility Index (mean difference 0.06 [90% CI 0.02–0.11]). Varying levels of agreement, ranging from slight to substantial (κ = 0.17–0.67), were demonstrated for the individual health domains. The order of telephone and patient-completed survey administration had no significant effect on results.

**Conclusions:**

Equivalent results are obtained between telephone and patient-completed administration for the VAS and Utility Index of the EuroQol Survey in people with advanced hip or knee osteoarthritis. The limits of agreement for the individual health domains vary which prevents the accurate interpretation of real change in these items across modes.

## Background

Health-related quality of life (HRQoL) is a key consideration in hip and knee arthroplasty. Pre-surgically, low HRQoL is one of the key drivers for hip or knee arthroplasty [1]. Post-surgically, hip and knee arthroplasties improve HRQoL substantially with large effect sizes evident in both the long and short term [2]. Together, these factors justify the use of HRQoL as an assessment tool in determining suitability for surgery and evaluating the success of surgery.

HRQoL is typically measured through a structured questionnaire. The Scientific Advisory Committee of the Medical Outcomes Trust has stated that these surveys should be evaluated according to eight psychometric criteria [3]:

- Conceptual and measurement model
- Reliability
- Validity
- Responsiveness
- Interpretability
- Respondent and administrative burden
- Modes of administration (MOA)
- Cultural and language adaptations

There are several HRQoL surveys currently being used to monitor HRQoL in hip and knee arthroplasty cohorts including the Short Form-36 and the Western Ontario and McMaster University Osteoarthritis Index [4]. Relevant to several arthroplasty clinical outcome registries (e.g. Arthoplasty Clinical Outcomes Registry, National; United Kingdom Patient Reported Outcome Measures; Swedish Hip Registry) is the EuroQol visual analogue scale (VAS) for joint pain and the associated Utility Index based on a 5-dimension health survey [5–7]. The EuroQol survey measures global patient-perceived health-related quality of life out of 100 on a 20 cm visual analogue scale (VAS). In addition, it measures HRQoL across five domains: mobility; personal care; usual activities; pain or discomfort; and anxiety or depression. In the 5 L version of the EuroQol Survey, each of the five domains is graded on a five-point scale ranging from no problems (1 point), slight (2 points), moderate (3 points), severe (4 points) or extreme/unable to perform (5 points). The resultant health state, represented by a five-digit profile, is then expressed as a Utility Index based on value sets derived for individual countries [8]. It is one of the most widely used HRQoL surveys internationally with translations available in 119 languages.

The current literature includes limited psychometric evaluation of the EuroQol survey while mode of administration has received little attention overall. Self-administered and interview-administered EuroQol questionnaires have been established to yield comparable results [9, 10]. Telephone and interview-administered questionnaires were similarly comparable [11]. Telephone administration has also been associated with better follow up and patient-reported scores as compared to mailed surveys [12, 13]. Notably, these studies were in cohorts of acute hospital admission, AIDS, rheumatoid arthritis, heart failure and cataract patients but not hip or knee arthroplasty patients; a group in which the EuroQol survey is commonly used. Furthermore, the telephone and self-completed modes of the EuroQol survey have not been directly compared.

The aim of this study was to determine whether the EuroQol survey provides equivalent responses for the VAS and Utility Index for the 5 EuroQol HRQoL domains given by telephone interview compared to paper-based form completion by the patient in an osteoarthritis cohort awaiting arthroplasty surgery.

## Methods

### Design and participants

Recruitment was conducted at a metropolitan teaching hospital (Nepean Hospital) in Sydney, Australia. The hospital is considered a high-volume arthroplasty service provider, performing an annual load of 290 hip and knee arthroplasty procedures at the time of the study. Patients 18 years of age or older and proficient in English were selected from the waiting list for primary knee or hip arthroplasty and invited to participate between May and August 2014. Exclusion criteria were patients scheduled to undergo arthroplasty less than a week after the earliest possible initial survey, or having joint replacement due to a fracture. Signed consent was obtained from all patients agreeing to participate. Recruitment was undertaken by physiotherapists experienced in the administration of the EuroQol survey. The study was approved by the institution’s human research ethics committee.

### Survey completion protocol

After agreeing to participate, each patient was administered the EuroQol survey via telephone and via a written, patient-completed form 1 week apart. The telephone interviews were scripted in a manner which replicated the original survey text and was performed by two trained personnel. As the visual analogue scale was not feasible over the telephone, a standardised verbal instruction was used to obtain a score of 0–100 and best replicate the effect of the scale. In contrast, collecting patient-completed responses via telephone did not refer to the survey text and was kept neutral by asking for their answer to the respective question number.

The 1-week interval between surveys was chosen as it balanced minimising the amount of time for the patient’s health status to change with allowing sufficient time that they would not be able to simply recall their previous answers [14]. To control for the effects of order of administration, patients were randomly allocated to completing the telephone or written survey first. A computer-generated sequence was provided by a researcher not involved in participant recruitment and allocation concealment was achieved by using consecutively numbered sealed envelopes, each containing the allocated order, which were opened individually upon each successful recruitment.

Those in the patient-completed first group filled in the survey unassisted at the clinic on the day of enrolment. A convenient time to call 1 week later was then arranged for the follow-up telephone survey. Those in the telephone first group received a telephone survey within days of providing consent to participate. The EuroQol survey was mailed 4–5 days after the phone interview so patients would receive it approximately 1 week after the initial survey, along with a telephone call for data-collection. Confirmation that they had hand-completed the survey was obtained prior to data-collection. Participants who had not completed their survey were asked to complete it on the spot. This method of survey return was chosen as it was intended to increase data completion by not relying on patients to post the completed paper surveys which has been associated with significant loss to follow-up [15].

### Statistical analysis

Ideally, the limits of variation in scores between the two modes of administration should be less than what is perceived to be the minimum important difference (MID) for changes in the VAS scale and the Utility Index, or equivalent to test-retest variation between the same MOA. In the absence of data indicating the MID for these indices for patients with osteoarthritis, we used data from a previously published study identifying the MID in a cohort of cancer patients [16]. A sample size of 58 people would be sufficient to find equivalence within ±7 for the VAS score, assuming a standard deviation of 20, correlation of 0.6 in responses between modes of administration, a significance threshold of 0.05 and 80% power. A sample of 29 participants would be sufficient to find equivalence within ± 0.11 for the Utility Index, assuming a standard deviation of 0.22, correlation of 0.6 in responses between modes of administration, a significance threshold of 0.05 and 80% power. Allowing for a loss to follow-up of 25%, the minimum sample required was 80.

The EuroQol questionnaire includes both categorical and continuous data. Continuous data from the VAS and summary Utility Index were subject to the Two One-Sided Tests (TOST) for equivalence. Equivalence bounds for the VAS and Utility Index were set at ±7 and ±0.11, respectively, in accordance with the EuroQol MIDs as stated above [16]. Significance for equivalence was set at *p* < 0.05. As the utility value set for Australia is yet to be determined, the United Kingdom value set was used for this study. Use of the UK values may affect the average calculated index values, but not affect the extent the two modes of administration are equivalent [17]. Bland-Altman plots of the data were also produced to illustrate the 95% limits of agreement (LOA) for both the Utility Index and VAS. These plots illustrate the range over which 95% of the paired data (scores from one method versus the other) vary in absolute terms [18].

Agreement between the categorical data was tested using Cohen’s kappa coefficient. The weighted Kappa scores used in the following analysis further distinguish between agreements or disagreements of varying gravity. This is achieved by weighting them differently to incorporate ratio-scaled degrees of agreement or disagreement [19]. Kappa scores range from −1 to 1, with higher scores indicating greater agreement. The typical kappa cut-offs are as follows [20]:

- ≤0: less than chance agreement
- 0.01–0.20: slight agreement
- 0.21–.040: fair agreement
- 0.41–0.60: moderate agreement
- 0.61–0.80: substantial agreement
- 0.81–1.00: almost perfect agreement

To determine the success of the randomisation process, we conducted chi-squared and two sample t-tests of patient characteristics between the groups subject to different orders of administration. A two sample *t*-test was used to determine if differences between modes was associated with order of administration.

The data analysis was generated using SAS Enterprise Guide Software, Version 6.1 of the SAS System for Windows (Cary, NC). Participants awaiting knee or hip arthroplasty were analysed together as the condition of interest was severe osteoarthritis and not the specific joint.

## Results

Ninety-three participants were screened for enrolment, three declined to participate and 15.6% (*n* = 14; 7 from each group) were lost to follow up, resulting in 76 complete datasets. There were no significant differences in patient characteristics between the telephone first and patient-completed first groups based on gender, age, joint type and side (Table 1). Follow-up interval (mean = 8 days, median = 7 days) and rates of loss to follow up were also not significantly different between the two orders of administration.

**Table 1:** Characteristics, mean telephone scores (_T_) and mean patient-completed scores (_P_) of the telephone-first group, patient-completed-first group and cumulatively

	Telephone First	Patient-Completed first	Total	*P-value*
Gender				
Female	20 (57%)	21 (43%)	49	0.15
Male	10 (37%)	17 (63%)	27	
Age (Years)				
Mean	68	68	68	0.98
Median	69	67	68	
Joint				
Knee	26 (53%)	23 (47%)	49	0.63
Hip	12 (44%)	15 (56%)	27	
Side				
Left	20 (48%)	22 (52%)	42	0.70
Right	15 (50%)	15 (50%)	30	
Bilateral	3 (75%)	1 (25%)	4	
Follow-Up Interval				
Days				
Mean (SD)	7.6 (1.3)	8.2 (1.6)	7.9 (1.5)	0.08
Median (IQR)	7 (2)	7 (3)	7 (1)	
Mean EuroQol Scores				
Mobility_T_	2.91	2.99	2.96	
Mobility_P_	2.89	3.45	3.17	
Personal Care_T_	1.71	1.95	1.87	
Personal Care_P_	1.80	2.35	2.08	
Usual Activities_T_	2.74	2.95	2.83	
Usual Activities_P_	2.74	3.17	2.97	
Pain & Discomfort_T_	3.17	3.30	3.25	
Pain & Discomfort_P_	3.14	3.74	3.46	
Anxiety & Depression_T_	1.57	1.69	1.67	
Anxiety & Depression_P_	1.91	2.05	1.99	
VAS Scale_T_	63.85	58.18	60.29	
VAS Scale_P_	59.94	60.94	60.34	

EQ-VAS scores were found to be equivalent between MOA (TOST *p* = 0.0013) within equivalence bounds of ±7. The VAS score was an average of 0.05 points lower (90% CI −3.76–3.67) when the survey was administered via telephone. Order of administration had no significant effect on differences in VAS scores between patient-completed and telephone survey modes (*p* = 0.20).

Utility Index was found to be equivalent between MOA (TOST *p* = 0.035) within equivalence bounds of ±0.11. This was an average of 0.06 points higher (90% CI 0.02–0.11) when the survey was administered via telephone. Order of administration had no significant effect on differences in Utility Index between patient-completed and telephone survey modes (*p* = 0.20).

**Fig. 1:**
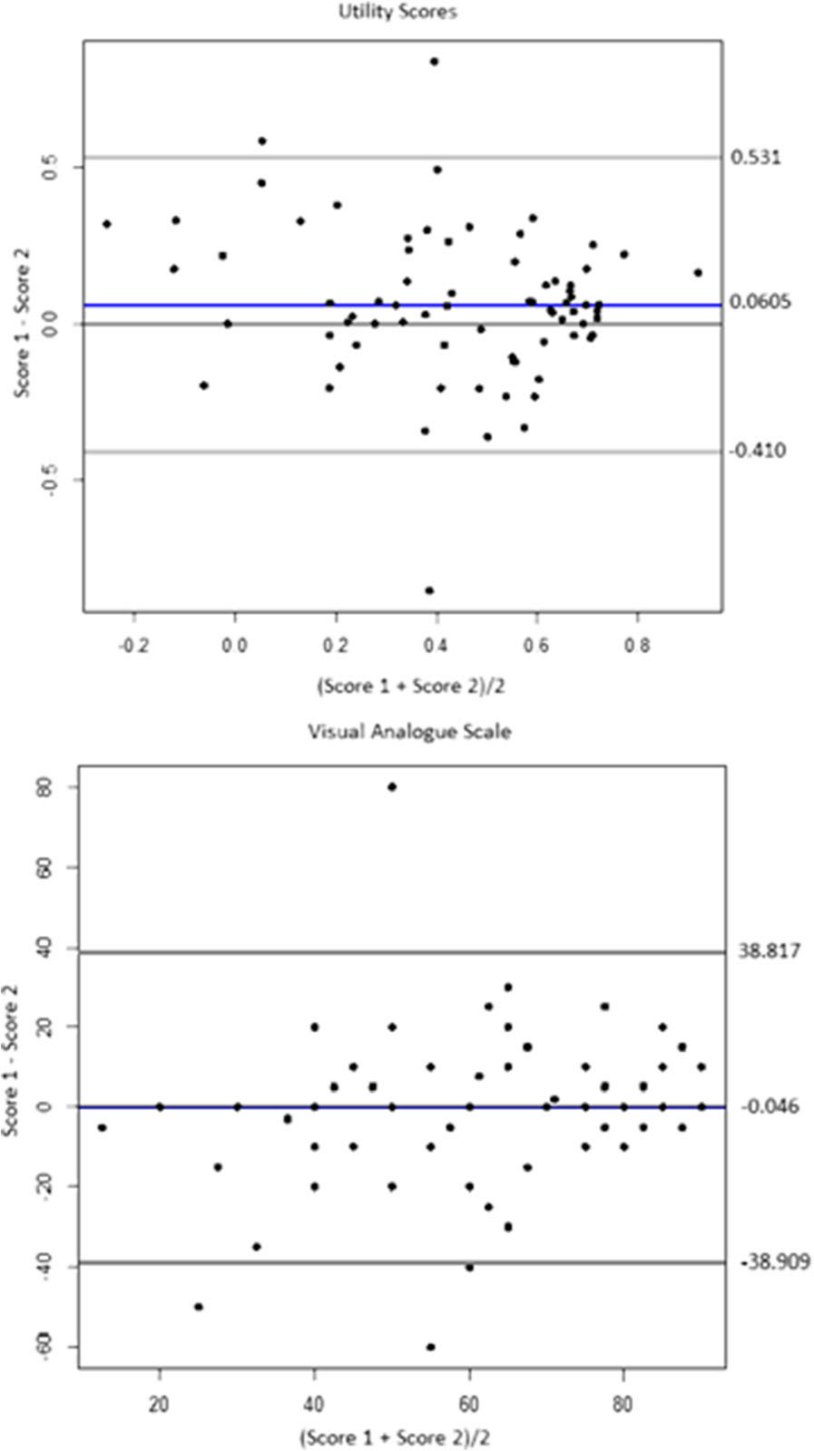
Bland Altman plots demonstrating the limits of agreement for utility and VAS scores. The mean difference and 2 standard deviations from the mean are respectively indicated by the *blue* and *grey* lines

The 95% limits of agreement for the Utility Index and visual analogue scale were relatively wide as shown in the Bland-Altman plot below (Fig. 1). This variation was consistent across the range of scores observed for both indices.

Weighted Cohen’s kappa coefficients reflected variable levels of agreement for the categorical data obtained via the two methods of administration. The personal care item exhibited substantial agreement (κ = 0.67) while the items for mobility and anxiety exhibited moderate agreement (κ = 0.45). Less agreement between the two MOA was found for the remaining items - usual activities (κ = 0.38) and pain (κ = 0.17).

## Discussion

Our study demonstrated equivalence between the telephone and patient-completed modes of administration for both the VAS and Utility Index of the EuroQol survey. This indicates that the telephone and patient-completed administrations can be used interchangeably if necessary. However, agreement between the individual domain scores varied between slight agreement and substantial agreement; indicating that interpreting changes in the individual questions across time if different modes are used is not recommended.

Interestingly, the between mode agreements observed here for the VAS and the Utility Index are similar to the week-to-week (test-retest) agreements of the VAS and Utility Index when completed by people with osteoarthritis using the same mode (patient-completed). We observed, testing people awaiting arthroplasty twice across a 1-week period, that the 95% LOAs for the VAS and Utility Index to be ± 29.4 and 0.4 respectively (unpublished data). Thus, it appears that the differences observed when changing between modes of the EuroQol are of similar magnitude to the differences observed when testing stability of the same method of administration over a short time span.

Establishing equivalence between modes permits survey administration through different modes without adjustment for the mode of administration. Furthermore, data collected by different but equivalent modes in different cohorts could be pooled for analysis. This broadens the scope for systematic reviews to compare studies that may have used different data collection methods.

The strengths and limitations of the study are acknowledged. We used a well-defined, homogenous sample of sufficient size to detect a difference as small as the reported MIDs for the VAS and Utility Index and the order of administration was randomised. The use of MID values based on those obtained using a cancer cohort may not be applicable to our cohort but MID values obtained using an orthopaedic cohort were unavailable at the time of the study.

## Conclusion

In conclusion, this study found that the summary Utility Index and VAS scales of the EuroQol survey were equivalent when obtained either by telephone or via a patient-administered mode. Despite equivalence in these components, greater variation is seen in the individual HRQoL items; indicating there could be no meaningful and accurate interpretation of change in the individual questions across time if different modes are used.

## Abbreviations

HRQoL: Health Related Quality of Life
LOA: Limits of Agreement
MID: Minimal Important Difference
MOA: Mode of Administration
TOST: Two One-Sided Test
VAS: Visual Analogue Scale
